# Disease control programme support costs: an update of WHO-CHOICE methodology, price databases and quantity assumptions

**DOI:** 10.1186/s12962-017-0083-6

**Published:** 2017-10-26

**Authors:** Melanie Y. Bertram, Karin Stenberg, Callum Brindley, Jina Li, Juliana Serje, Rory Watts, Tessa Tan-Torres Edejer

**Affiliations:** Department of Health Systems Governance and Financing, World Health Organization, Avenue Appia 20, Geneva, Switzerland

## Abstract

**Background:**

Estimating health care costs, either in the context of understanding resource utilization in the implementation of a health plan, or in the context of economic evaluation, has become a common activity of health planners, health technology assessment agencies and academic groups. However, data sources for costs outside of direct service delivery are often scarce. WHO-CHOICE produces global price databases and guidance on quantity assumptions to support country level costing exercises. This paper presents updates to the WHO-CHOICE methodology and price databases for programme costs.

**Methods:**

We collated publicly available databases for 14 non-traded cost variables, as well as a set of traded items used within health systems (traded goods are those which can be purchased from anywhere in the world, whereas non-traded goods are those which must be produced locally, such as human resources). Within each of the variables, missing data was present for some proportion of the WHO member states. For each variables statistical or econometric models were used to model prices for each of the 194 WHO member states in 2010 International Dollars. Literature reviews were used to update quantity assumptions associated with each variable to contribute to the support costs of disease control programmes.

**Results:**

A full database of prices for disease control programme support costs is available for country-specific costing purposes. Human resources are the largest driver of disease control programme support costs, followed by supervision costs.

**Conclusions:**

Despite major advances in the availability of data since the previous version of this work, there are still some limitations in data availability to respond to the needs of those wishing to develop cost and cost-effectiveness estimates. Greater attention to programme support costs in cost data collection activities would contribute to an understanding of how these costs contribute to quality of health service delivery and should be encouraged.

## Background

Estimating health care costs, either in the context of understanding resource utilization in the implementation of a health plan, or in the context of economic evaluation, has become a common activity of health planners, health technology assessment agencies and academic groups. A critical component of health care costing is the estimation of resource utilization linked to activities of a disease control programme aimed at supporting the quality of delivery or encouraging the uptake of a specific set of services, but which do not occur as part of direct service provision. Johns et al. [1] defined programme costs as those “incurred at the administrative levels outside the point of delivery of health care to beneficiaries” and included categories of costs such as personnel, media and utilities. Other studies report on “above-facility-level” costs (e.g., Galarraga et al. [2]) as any relevant costs occurring at a higher administrative level than the facility (i.e., district/provincial/national program management). Here, we expand the definition provided by Johns et al. and define programme support costs as costs that (a) reflect a set of activities that are specific to, and designed in relation to, a group of identified health interventions and/or technologies^1^ usually for the same disease/health condition and aimed at improving the quality of delivery or encouraging the uptake of services, and (b) incurred at an administrative level that is outside the point of delivery.

We define programme support costs are those which occur outside of direct service delivery, such as the drugs and tests associated with an intervention, but are not shared health system costs such as supply chain and infrastructure. Programme support costs are for those activities taking place at the national, district and province level which are directed towards enhancing the quality of a programme, for example training, supervision visits and monitoring and evaluation. A programme is considered a set of interventions which contribute to the prevention and control of a common health outcome—for example, HIV, maternal and newborn health, non-communicable diseases.

Since its conception in 1998, the WHO-CHOICE programme has advocated the use of an ingredients based approach identifying all resources required to deliver a health care intervention, quantifying the resource requirements *q* and assigning a price to each resource *p*. The multiplication of *p* and *q* then gives us the cost.

WHO-CHOICE takes the costing perspective of “the health system”, by which is meant the ensemble of actions and actors whose primary intent is to improve human health. CHOICE therefore includes all direct, market-valued costs, whether public or private, that are required to deliver the intervention, regardless of payer. WHO-CHOICE does not account for non-monetary patient contributions such as travel time, time off work, or lost income—nor do we account for costs outside of the health system, such as the cost of social services whose aim is not primarily health oriented. So the costing perspective of CHOICE is broader than the health sector per se, and is health system focused according to accepted international definitions of the health system. Other sector costs (e.g. legislation) are included to the extent that they are a direct component of the intervention that is intended to improve human health.

Intervention costs are divided into patient and programme levels, and where relevant complemented by health system costs. The WHO-CHOICE programme cost database is primarily set up to support generalised cost effectiveness analysis (GCEA), but it is also used for other purposes such as the production of global resource needs assessments and investment cases, both of which require processing data for multiple interventions using a standardised format for *p* and *q*. WHO-CHOICE GCEA includes patient level costs incurred at the point of delivery, i.e., medicines, tests and health facility visits (including human resources); as well as programme support costs which reflect the additional activities required to run a disease control programme, such as administration, monitoring and evaluation, supervision, legislation, training and law enforcement (Table 1). The scope is defined by the need to do an economic evaluation of single interventions or combinations of interventions, therefore only intervention-specific resources are considered for the GCEA.

**Table 1:** Scope of programme support costs vis-a-vis other cost categories within two types of WHO-CHOICE analysis

Area of work at global level	Economic evaluation e.g. WHO CHOICE	Global resource needs assessments e.g. SDG health price tag
Costs directly related to individual intervention delivery (patient costs)	Medicines, diagnostic tests, other consumables	Medicines, diagnostic tests, other consumables
	Behaviour change communication	Behaviour change communication
	Health facility visit unit costs, incorporating health system costs	
Costs related to delivery of a health programme e.g. NCD, maternal and child health (programme costs)	Personnel	Personnel
	Materials and supplies	Materials and supplies
	Media	Media
	Transport	Transport
	Equipment	Equipment
	Maintenance	Maintenance
	Utilities	Utilities
Costs related to health system functions (system costs)	Supply chain	Supply chain
		Health workforce
		Health infrastructure and equipment
		Governance
		Health financing
		Health information systems

Within WHO CHOICE, programme costs are included in GCEA as the comparison of single interventions is only the starting point. In constructing an expansion path, the programme costs will change as more interventions are added to the pathway, creating a difference in marginal costs between each additional intervention. The only time when programme costs would not be relevant is in a marginal decision between two interventions which act within the same programme area—for example a choice between metformin and sulfonylurea for treatment of diabetes, where only the costs of the drugs differ. Programme costs are important as they are part of the full cost of delivering the intervention. Without their inclusion the cost-effectiveness ratio calculated could be misleading.

A disease control programme within this context is a set of interventions addressing a common need (epidemiological or population group). Disease control programmes may differ significantly in their cost drivers, with those aiming for behaviour change requiring more programme-level costs and less direct patient-level resources, e.g., tobacco control programmes. Similarly, interventions introducing new technologies may require more intense upfront investments in specific components such as training and supervision. Examining programme-level costs is therefore essential to understand the full resource need. In 2005 WHO-CHOICE produced a global price database to facilitate the estimation of programme costs, with a reference year of 2000, along with generic costing templates with quantity assumptions. While quantity assumptions are standardised, the database provides prices at the level of WHO region^2^ and countries for a range of goods such as salaries, utilities, transportation, telecommunications, media and consumables. Many analysts still rely on the WHO-CHOICE price databases and estimates of quantity assumptions for programme cost estimation.

In 2013 a process to update the WHO-CHOICE price database, to a reference year of 2010, along with a review of the quantity assumptions, was started. This paper reports the data sources and methodologies used in the update of the price databases, the new quantity assumptions and suggested scaling factors for programme support costs based on the number and coverage of interventions delivered by a programme.

This paper seeks to achieve two aims: (i) to describe the methods used to derive updated estimates for prices of inputs that go into programme costs; and (ii) to define the scope of a set of programme costs that go with a WHO-CHOICE CEA application, and describe new quantity assumptions for the typical WHO-CHOICE CEA application, including newly developed scaling factors.

## Methods

The starting point of the 2010 update of the price database was a review of the 2000 database, including data sources and methodology. Table 2 replicates the cost categories reported on in the 2003 publication [1]. This article reports on data used for the estimation of the data points in Table 2 with the exception of personnel costs which are reported separately. In an advance from the earlier methodology, prices are now reported at the country level rather than the regional level.

**Table 2:** Cost categories required for programme costing

A. Recurrent cost	
A.1 Personnel	Personnel time allocated to each intervention is assessed from time spent by those personnel in other interventions. Personnel time used in the start-up and post start-up periods is expressed in person-months
A.2. Materials and supplies	Materials and supplies in terms of the quantities used for the programme. Examples are office supplies (e.g. stationary) that are used by the programme
A.3. Media operating costs	Media inputs such as radio or television time, leaflets or posters are provided in terms of their unit of measurement (e.g. minutes for radio, or quarter page ads in newspapers)
A.4. Transport operating costs	Transport is measured in terms of total kilometres travelled per mode of transport
A.5. Equipment operating cost	In cases where equipment is rented, the amount of equipment and the duration of rental (in months) are reported
A.6. Maintenance	Maintenance costs are listed as a percentage of annual costs
A.7. Utilities	The amounts of utility items allocated to the programme are listed here. Examples of utility items are electricity, gas, and water. The allocation of the quantities used by the programme is based on the square meter surface area used by the programme, then applying any further allocation needed if the space is shared with other programmes
A.8. Others	
A.8.1. Rented buildings	In case where buildings are rented, both the total square meter surface area of the buildings and the duration of rental (in months) are used
A.8.2. Per diems and travel allowances	The types of personnel who are entitled for per diems and travel are listed. The types reflect the activity they are involved in, e.g. trainers, trainees, support staff in meetings, participants of meetings, supervisors visiting health facilities etc. Reported by the number of days per type of personnel
A.8.3. Miscellaneous items	Any other category of recurrent resources used that is not provided in the list are reported here by identifying the item and the quantities used
B. Capital costs	
B.1. Building	Spaces used by the programme are reported in terms of the total square meter surface area allocated to that programme, i.e., if a space used by the programme is shared with other activities, the share of the space used for the programme under study is estimated and the value is entered here
B.2. Transport	The number of modes of transport used by the programme is listed here. If they are only partly used, the estimated share of their use are entered
B.3. Equipment and implements	The quantity of office equipment, storage and distribution, maintenance, cleaning and other capital equipment are reported here. If they are only partly used, appropriate allocation is made, using the same allocation factors used for building space
B.4. Furniture	See point B.3 above
B.5. Other capital costs	This section is used to report any other capital resources used by the programme

### Estimation of price databases

For each data point, we searched the internet to identify global price databases covering as many WHO member states as possible. This section reports, for each data point, the data reported in the WHO-CHOICE price database and the methodology used to extract data and estimate missing values.

#### WHO member state meta-data

The database contains the following general information on each WHO member state:

- WHO region.
- Population.
- Total GDP.
- World Bank income level classification.
- USD exchange rate and GDP deflators.
- Number of administrative divisions within each member state.
- Number of certain types of healthcare facilities including health posts and hospitals.
- Density of health care facilities per 100,000 people.

The general information including population, GDP and price deflators for each WHO member state is sourced from WHO’s Global Health Expenditure Database [3]. The number of administrative divisions is taken from the website *Statoids: Administrative Divisions of Countries* [4]. The terminology first sub-national division and second sub-national division is used to allow for differences in nomenclature for administrative divisions. These sub-national divisions may differ considerably in terms of geographical size and population depending on the country. The number and density of health care facilities is taken from WHO’s 2013 Country Survey on Medical Devices and Health Care Facilities [5], and may include both public and private sector facilities. Unlike elsewhere in this update, the information on healthcare facilities is based on data for the year 2013 rather than 2010, due to the year of data availability. The year on which the information on administrative divisions is based also varies. This information, which is sourced from the website *Statoids: Administrative Divisions of Countries*, uses the most recent national survey and usually dates to sometime in the past 5–6 years. Missing data were imputed using iterative robust model based imputation [6].

#### Travel allowance and per diem rates

The database contains for each WHO Member State a daily subsistence allowance and accommodation rate in USD at 2010 prices for the capital city and a lower and upper range for other parts of the country where available. The average daily cost of living rates for a number of cities was calculated from an online database of cost of living values on numbeo.com [7]. These data points were compared to travel allowance and per diem rates sourced from the International Civil Service Commission (ICSC) DSA Circular Report for July 2010 [8]. The local cost of living rates from numbeo approximated 20% of the ICSC travel allowance and per diem rates, thus a scaling factor of 0.2 was applied to the ICSC rates to approximate local per-diem values. A further 5% of the value was added to account for economic costs associated with meetings, such as room hire fees. The ICSC database provides per diems for all WHO member states, thus no estimation of missing values was required.

#### Vehicle and transportation costs

The database contains new vehicle costs for several modes of transport used in the field for health interventions, including sedans, four-wheel drives, utility trucks, motorcycles, mini-buses and refrigerator vehicles. The database also includes their approximate per kilometre operating costs. Prices and vehicle specifications for a set range of vehicles were sourced from UNOPS procurement website, UN Web Buy [9]. These prices provided are roughly illustrative of the market prices faced by medium-sized international organisations or governments of low-income countries. Where available, *ex*-*stock* prices were selected over *ex*-*factory* prices since they are closer to the final price paid by the buyer. Specifically, they include free carrier charge to move the vehicle to a port ready for export. Freight, insurance and customs duties to export the vehicle are not included in the price because they are likely to vary significantly between destination countries. Operating cost per kilometre is estimated as the sum of the following three expenses.

1. Fuel economy = fuel price/L × fuel consumption/km.
2. Tyre cost/km = cost of four new tyres/average lifespan of tyre in km.
3. Cost of basic maintenance/km (0.5% of new vehicle value/20,000 km).

Fuel price was sourced from the World Bank Indicator database [10], fuel consumption level/km from the US Environmental Protection Agency [11], and tyre costs and tyre lifespans were estimated with reference to automotive websites such as Michelin and Dunlop. Missing data were imputed using Iterative Robust Model Based Imputation [6].

#### Cold chain storage and distribution

The database contains the costs of an assortment of cold-chain equipment and devices relevant to health interventions. The list of cold-chain equipment and devices was compiled based on the general requirements of immunisation programmes, which use cold-chain storage and distribution extensively. As such, these products can also be applied to other disease control programmes requiring cold-chain management. The list was prepared in consultation with the WHO prequalification team (PQT) and with reference to:

- WHO (2002) Guidelines for Estimating Costs of Introducing New Vaccines into the National Immunization System [12].
- WHO’s Performance, Quality and Safety (PQS) Catalogue [13] and,
- UNICEF’s Supply Catalogue [14].

These cold-chain products are relevant in a variety of contexts, such as different climates and varying health care infrastructure quality. Where a product, such as a cold room or cold box, might have several models varying in characteristics such as size, a single representative model, which is of high quality and value for money, is listed for simplification. This choice was made in consultation with WHO PQT. As of December 2013, all of these products were pre-qualified under WHO’s PQS system. The prices of the specified models were collected from WHO’s PQS Catalogue and UNICEF’s Supply Catalogue. The prices in these catalogues are listed in principal international currencies such as USD, EUR and CHF. In most cases, a price year was also included which varied from 2007 to 2013. Non-USD prices were converted into USD using the WHO’s Global Health Expenditure Database exchange rate for years up to 2011 [3]. For the price years 2012, 2013, and those that were unknown, the World Bank’s average exchange rate for 2012 was used. When different prices for the same product were listed in the WHO and UNICEF catalogues, the WHO listed price was used. The inclusion of specific products in this list is for the purpose of more accurately estimating the costs of inputs using a consistent methodology across programmes. In no way does this imply an endorsement or promotion of the companies making these products.

#### Power generation and utility prices

The database contains the costs associated with power generation and general utilities for electricity (via mains power, diesel generators and solar panels) and water.

Data on the price of electricity supplied by mains power was sourced from the International Energy Agency’s (IEA) quarterly publication *Energy Prices and Taxes*, 3rd quarter 2011 [15]. Given that the price of electricity faced by households and industry can be significantly different, a general price was obtained by taking an average of the two values. Prices for 2010 in USD/kWh for OECD countries were taken from Part II. F. Tables 21 and 22. Prices for 2010 in USD/kWh for selected non-OECD countries were taken from Part III B. Tables 18 and 19. Note that this data does not use PPP. For countries that did not have data for 2010, the most recent year was taken and an approximate 2010 price was calculated using energy price indices from Part II A. and Part III A. For further information on the methodology of collecting and calculating these electricity prices, see the IEA publication [15]. Data on the price of electricity supplied by diesel generators and solar power systems was sourced from UNICEF’s Supply Catalogue [14].

The electricity dataset contained data for only 26% of WHO Member States, thus it was not suitable to be included in the Iterative Robust Model Based Imputation. Instead, a simple linear regression using GDP/capita as the explanatory variable was used to estimate missing values.

Data on water prices was sourced from the Global Water Intelligence (GWI) Survey, conducted in August, 2011 [16]. The GWI survey includes water and waste-water prices from 309 cities in 106 countries and is the most comprehensive single source of information on water prices. The price of water/m^3^ in the local currency is calculated based on the use of 15 m^3^ of water/month and converted to USD. For further information see the GWI survey’s methodology. A water price for each country in the GWI survey was compiled based on the average price of water in its surveyed cities. This national price for 2011 given in USD was converted into a 2010 price using the survey’s available information about the previous year’s price increases. The dataset covered 52% of WHO Member States, and the Iterative Robust Model Based Imputation was used to estimate missing values.

#### Telecommunication data

The database contains 14 telecommunications variables:

- Fixed (wired) broadband connection charge in USD.
- Fixed (wired) broadband monthly subscription charge.
- Monthly subscription for business telephone service (USD).
- Installation fee for business telephone service.
- Price of a 3-min local call to a fixed-telephone line (peak rate),
- Price of a 3-min local call to a fixed-telephone line (off-peak rate),
- Mobile prepaid—1-min local call (off-peak, on-network).
- Mobile-prepaid—1-min local call (peak, on-network).
- Mobile prepaid—1-min local call (peak, to fixed).
- Mobile prepaid—1-min local call (off-peak, to fixed).
- Mobile prepaid—price of SMS (on network).
- Mobile prepaid—1 min local call (off-peak, off-net).
- Mobile prepaid—1 min local call (peak, off-net).
- Mobile prepaid—SMS (off-net).

Data was obtained from the International Telecommunications Union (ITU) Information, Communication and Technology (ICT) indicators database [17], with data for the period 2008–2012 used in this analysis. Between 13 and 42% of values were missing for each variable. Multiple imputation by chained equations was used to fill missing values, so that data was available for each country. An econometric pricing model was then developed for each telecommunication variable, using a system generalized method of moments approach for dynamic panel models. The data presented in the WHO-CHOICE dataset are modelled data from the econometric pricing model, and thus may differ from those presented in the ITU dataset. Further information on the estimation of this dataset is available in the Masters Thesis [18].

#### Office supplies

The database contains costs associated with office furniture and supplies such as stationery and basic ICT hardware. The list of office supplies, ICT equipment and furniture was prepared by considering the basic resources required to operate a small office. If necessary, these resources were treated as tradable goods that could be imported at a cheaper price than what is locally available. The costs of the office supplies and furniture were sourced from the 2014 Global Supply Catalogue of the US General Services Administration (GSA), which is accessible to US government agencies and eligible NGOs and International Organisations including the United Nations [19]. Using this source has the following benefits:

- Prices are based on a competitive procurement process and economies of scale.
- Reliable supply of goods at stated price is guaranteed.
- Large range of goods are costed from a single, up-to-date source.
- Prices listed included freight and insurance to destination port.

#### Advertising

The average costs of advertising could not be found for any set of countries despite an extensive search. Most information publicly available pertains to estimates of the value of different segments of the advertising industry. That is, the revenue that it raises. The key publication on this is the PricewaterhouseCoopers (PWC) annual report, Global Entertainment and Media Outlook [20].

In 2000 an econometric model estimating advertising prices (per 30 s of TV or radio time, per half page newspaper advertisement and per A4 page printed) as a proportion of GDP/capita was created. No new data was available to contribute to an updated econometric model, thus this model was applied to 2010 GDP/capita by country to estimate updated media prices.

### Estimation of quantity assumptions

This section outlines standard quantities of inputs assumed to be required to implement a programme with ten health interventions at full coverage (i.e., all those in need of the interventions would receive them).

#### Administration requirements

Despite recent strong focus on planning for Human Resources for Health, there is scant quantitative evidence on staffing requirements. At a minimum, WHO has estimated 4.45 “health workers” (doctors, nurses and midwives) per 1000 population will be required by the end of the SDG period [21]. However, these ratios do not touch on broader policy, administrative, training etc. related to health personnel. For staff requirements at the programmatic level (administration, management etc.) we use a combination of assumptions from a costing study carried out for the 2005 World Health Report and a 2008 NCD costing study undertaken within WHO [22, 23]. Both studies used expert groups to gain consensus around staffing requirements and ratios.

Materials and supplies (e.g. consumables such as stationary) build off of previous WHO-CHOICE estimates with some important updates. Assumptions around computer storage devices have been updated in line with current technology. However, computer and printing equipment assumptions have remained constant, including useful life of devices, ratio of printers and photocopiers required per person. Also important to note is that computer prices have not increased in line with inflation. Again, there were no quantitative references available to base the quantity assumptions for this section of the programme support costs; therefore expert assumption and consensus was the basis for the quantity assumptions.

#### Utilities

In line with the previous WHO CHOICE methodology, quantity assumptions for electricity are based on 3 people occupying a work space using an average of 64 kWh/month. Water consumption is assumed at 10 m^3^/office of 3 people/year. Telephone calls are assumed as 2 calls/person/work day [1].

#### Information, education and communication (IEC)

Radios are present in at least 75% of households in developing countries [24]. Radios account for 86% of listening to an audio platform, with the remainder being web based or satellite radio [25]. Online searches indicate that in order to impact individuals, an advertisement needs to be heard between 5 and 7 times [26]. In order to calculate how many times to run an advertisement to reach listeners with this frequency, the following formula is proposed [27].

$${\text{Quarter}}\;{\text{hour}}\;{\text{listeners}}/{\text{Cumulative}}\;{\text{weekly}}\;{\text{listeners }} = {\text{ X}}$$

$${\text{Frequency of hearing required}}/{\text{X }} = {\text{ number of times to run the spot}}$$

In order to use this data for our quantity assumptions, we require data on listenership for each of the regions included in our analysis, or average listenership in low and middle income countries.

Radio ownership and listenership was found for a selection of African countries. Similar data have not been identified for other global regions, so the assumptions are based on Africa alone. The amount of time/day that each person listens, on average, was not identified. Calculations for the number of advertising minutes required are based on the assumption that 56–78% of people listen to the radio daily [28]. We assume each person listens for at least 60 min/day, and that there were 12 h/day within which an individual could listen to the radio. Between 87 and 90% of people own a radio.

Thus a radio advertisement of 30 s, played 82 times/week would achieve the required listenership over the 30 day schedule as recommended in the marketing literature.

The same information applies to television as radio advertising—individuals need to see an advertisement five times to remember it. Television ownership in low and middle income countries is lower than radio ownership; however, this statistic has large variation across regions. We assume the same intensity is required but that the proportion of the population reached is lower than for radio advertisements due to lower ownership.

#### Supervision

Supervision is carried out for the programme as a whole. The staff carrying out supervision visits are those whose wages are already accounted for in the human resources segment. We assume each province, district and facility is visited twice per year for 2 days at a time.

#### Training

At the programme level the model assumes that each programme conducts one in-service/refresher training at the provincial level per year, and one per district every 3 years. A training of trainers workshop is required each year. Specific interventions, such as newly implemented interventions, may require more intense training but we assume these costs to be analysed as part of the intervention implementation cost.

#### Vehicles

At the national level we assume an all-terrain vehicle is required for each programme. We assume each province requires one heavy-duty 4 × 4 per programme, and at the district level 3 motorcycles are needed. These assumptions were validated with national planners in a non-random, purposive selection of countries.

### Scaling of programme and delivery costs to account for economies of scale and scope

#### Scaling programme support costs by number of interventions

We make the assumption that a programme running at full capacity can support the implementation of ten interventions at full coverage. This assumption is based on the experience of the authors working in countries developing costed national health plans. No literature was found in support of such an assumption, thus country users of the methodology are strongly encouraged to evaluate the appropriateness of this assumption in their setting. If running at a lower capacity, the human resources, vehicles and office running costs are reduced. We assume that to implement a single intervention, 30% of these resources would be required, with a linear increase to 100% resource requirement for ten interventions. Following the 10th intervention a marginal 3% increase is added for each additional intervention. Costs for training and supervision are assumed to be constant regardless of the number of interventions delivered.

#### Scaling programme support costs by coverage (current, 50,80,95)

We assume that human resource and office running costs remain constant regardless of the coverage level of interventions delivered by the programme however are influenced by the quantity of interventions delivered by the programme. In-service training and training of trainers are scaled to coverage. This assumes clients seeking care will be seen by the right trained health worker when they access health services—e.g., as in a geographically targeted scale-up. However, development of training programmes and materials remains a fixed investment at 100% regardless of coverage level. Supervision visits to staff are scaled to coverage (i.e. fewer visits are required for lower capacity, as fewer patients are seen). All other costs are assumed to remain at 100% regardless of the coverage level.

#### Scaling costs of drugs to account for supply chain costs

Supply chain costs per se are not theoretically covered under programme support costs, but the WHO-CHOICE model for GCEA applies a mark-up ratio in order to capture these *health systems* costs as part of this economic evaluation. We assume a constant supply chain multiplier regardless of coverage level, as we were not able to find adequate evidence of increasing logistical costs as coverage increases. We apply the multipliers below based on data reported for countries which are deemed to have a fairly well functioning systems [29]. Overall commodities (other than ITNs): apply 13% mark-up.

- ITNs: apply 26% mark-up (as they are more bulky).
- Cold chain costs: apply an additional 6%.

## Results

Table 3 shows a summary of data sources and methodologies used in the estimation of the programme costing database. The full database is contained in Annex 1 to this paper, and available for download online at http://www.who.int/choice/costs/en. The data is presented at the country level for 194 WHO member states.

**Table 3:** Summary of data sources and methodology for missing value imputation

Variable	Source	Method for estimating country level data
GDP	International Monetary Fund [30]	n/a
Population	United Nations Population Division [31]	n/a
Exchange rate	WHO Global Health Expenditure Database [3]	Iterative Robust Model Based Imputation [6]
Administrative divisions	Statoids: Administrative Divisions of Countries [4]	Iterative Robust Model Based Imputation [6]
Healthcare facilities	WHO 2013 Country Survey on Medical Devices and Healthcare Facilities [5]	Iterative Robust Model Based Imputation [6]
Travel allowance and per diem	International Civil Service Commission DSA Circular Report for July 2010 [8]	Comparison to cost-of-living index and reduction to 20% of ICSC rate
Vehicles	The United Nations Office for Project Services [9]	n/a
Fuel prices	World Bank [10]	Iterative Robust Model Based Imputation [6]
Cold chain	WHO Performance, Quality and Safety (PQS) Catalogue [13] UNICEF’s Supply Catalogue [14]	n/a
Generators	WHO PQS Catalogue [13]	n/a
Electricity	International Energy Agency’s (IEA) quarterly publication Energy Prices and Taxes [15]	Linear regression
Water	Global Water Intelligence (GWI) Survey for 2011 [16]	Iterative Robust Model Based Imputation [6]
Office supplies	2014 Global Supply Catalogue [19]	n/a
Information and communication technology	International Telecommunications Union [17]	Multiple imputation by chained equations [32, 33], System Generalized Method of Moments [34]
Media		Linear regression

The main imputation dataset contained 24 variables, of which 9 had complete data available and 15 had varying levels of missingness. The first step in the analysis was to correlate all variables with missing data against the 9 complete observations, to test the relationship of the missing variable against the complete variables. This indicated that there was a lack of consistent correlation against the 9 complete variables, such as GDP/capita, population size and area. This implied that the use of linear regression or conditional mean to impute variables was not an ideal estimation method. Of the options available related to missingness, missing completely at random was considered too strong an assumption as this is rarely the case in real world data, and missing not at random was not felt to be a valid assumption as there was no systematic appearance to the missing data. Thus missing at random (MAR) was assumed.

Following imputation, the upper and lower bounds along with the mean value of the observed data was compared to the completed dataset for each variable. We observed consistency between the observed and completed datasets for all three metrics, indicating the imputation method is acceptable. In a further test, we checked the conditional distribution assumption of each variable with missing values—each variable containing missing values is normally distributed conditional on all the other variables. To test this, we regressed the observed part of one variable on the corresponding values of the other variables in the completed dataset. The residuals for all the variables that went through this test turned out to be approximately normal, indicating our assumptions had been met.

The price database is primarily used for two purposes: WHO-CHOICE generalised cost effectiveness analysis (GCEA) and the production of global resource needs assessments, both of which require extensive datasets to be generated and processed for multiple countries. A specific requirement is standardisation across interventions, which is provided through a programme costing calculation Excel workbook. This incorporates the price database, along with the quantity assumptions and scaling factors outlined. Users can select a country and are provided with generic estimates for the country of interest, with the possibility to modify all prices and quantity assumptions. The file differentiates between ongoing costs (expected to occur each year to maintain a current level of service) and set-up costs (costs that are associated with the introduction of a new programme or with the scaling up of an existing programme). This calculation workbook is used in the computation of programme support costs for WHO-CHOICE GCEA analyses. It also maps to the programme costing menus in the OneHealth Tool (http://who.int/choice/onehealthtool/en/), enabling compatibility between the two tools.

Note that this workbook is designed for the express purpose of calculating programme support costs for use in economic evaluation. Applying the calculation workbook as used in the computation of programme support costs for WHO-CHOICE GCEA analyses for a global price tag would not be appropriate since it computes economic costs, and not financial costs. Thus, capital investments are annuitized within the calculation workbook whereas the WHO global price tags do not annuatise such costs since doing so would not adequately illustrate financial outlays for capital purchases, which may form a considerable share of start-up costs.

**Fig. 1:**
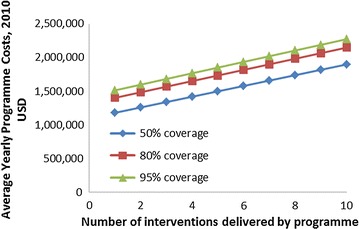
Programme support costs by coverage level, for a hypothetical country in Asia of 20 million people, with a GDP/capita of approximately $2300 USD

For a hypothetical country in Asia of 20 million people, with a GDP/capita of approximately $2300 USD, Fig. 1 shows the programme support costs as calculated using this approach for a generic programme, including the scaling based on number of interventions and coverage level. The programme support costs differ between diseases due to intensity of training, supervision and media campaigns.

**Fig. 2:**
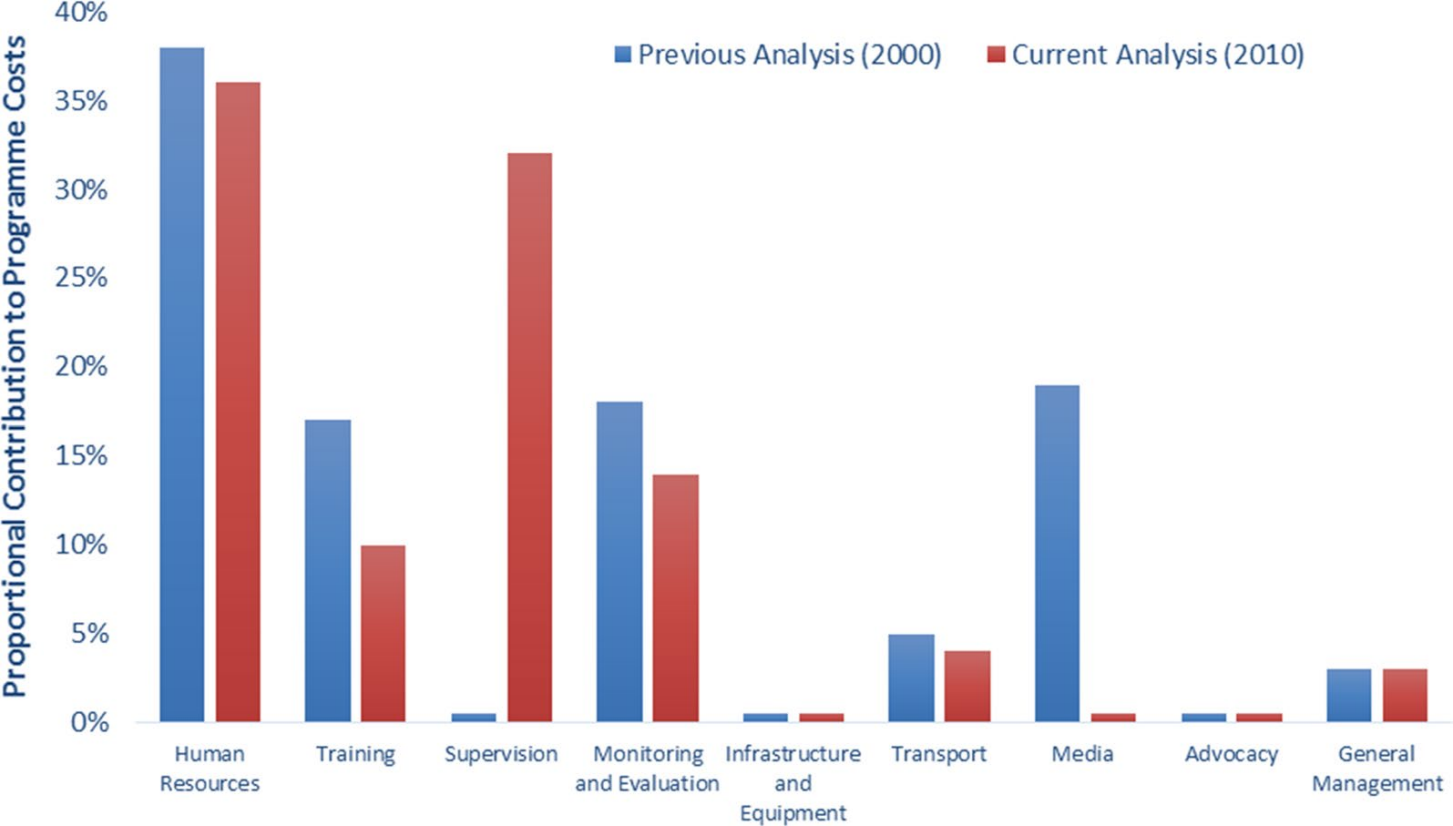
Share of total programme costs contributed by major categories, for a hypothetical country in Asia of 20 million people, with a GDP/capita of approximately $2300 USD

Comparing the analysis of programme support costs to the previous analysis from 2000 (expressed in GDP-inflated 2010 USD), both similarities and differences are present (Fig. 2). Firstly, programme specific human resource costs have remained consistent at almost 40% of the overall cost. Due to their major contribution, and the expert opinion used to define quantity assumptions, a sensitivity analysis was undertaken on the quantity data. If human resource requirements are half of that estimated, the contribution to overall costs would decrease to approximately 20%, whereas doubling the quantity needs would increase human resources to 55% of total programme support costs. Media costs have reduced significantly, previously representing almost 20% of total costs and now 0.5%. This difference is driven by a conceptual change in WHO-CHOICE methodology. In 2000, media costs associated with a behaviour change communication intervention—i.e. those which have a direct impact on health outcomes—were included as programme costs, now they are considered intervention specific costs for GCEA and calculated as such, for example so that a mass media campaign to prompt smoking cessation is regarded as an individual intervention. We made this change to foster “fair comparisons” when undertaking economic evaluation of media-intensive behaviour change interventions compared with other interventions which include only basic programme advocacy costs. Media costs now included in programme support costs are for basic advocacy purposes only. Monitoring and evaluation costs have dropped in overall share, however, supervision has increased sharply. Previously, supervision costs were incorporated into human resource and transportation costs. With a growing body of evidence that highlights the important contribution of supervision to programme success, it is now being calculated explicitly. These types of changes in conceptual thinking, as well as a combination of changing prices and quantity assumptions, make drawing overall conclusions on comparability between the databases and analyses uninformative.

## Discussion

All data modelled by WHO-CHOICE is available for download from the website http://www.who.int/choice and is available as an Annex to this paper. A tool to undertake programme costing, incorporating the quantity assumptions in this paper is also available. Estimates are specific to each country and should be considered as normative, global costs that have not been validated by countries.

This work represents the most comprehensive update of the WHO-CHOICE programme costing prices and quantity assumptions since they were first published in 2003. Between 2000 and 2010, global prices have undergone substantial changes, and continuing to inflate prices from 2000 to the present ignores underlying changes in cost structures that can be complex. Given the substantial contribution programme costing can have on the total resource allocation for implementing health care interventions, ideally data such as these would be continually updated to keep track with these changes in the global economy.

Compared to the 2000 estimates, a greater amount of data was able to be sourced from online databases, with econometric or statistical models used to generate missing values. However, for some variables, such as media and construction, freely available global databases do not exist. The development of a global database reporting prices and quantity assumptions collected in costing studies would be a helpful contribution to future updates of this work.

Despite major advances in the availability of data since the previous version of this work, there are still some limitations in data availability to respond to the needs of those wishing to develop cost and cost-effectiveness estimates. Firstly, global price databases on prices present missing values for up to 60% of countries, with many of the gaps seen in low income countries, where the greatest need for accurate cost data is present (Table 4). This increases the uncertainty in estimating country specific prices and reduces methodological consistency across the data points within each cost category. Secondly, not all data points that would be desired are available in accessible databases, with high access costs associated, thereby limiting the ability of researchers to access the data. Thirdly, limited information exists regarding quantity assumptions of human resources needed to support the implementation of health care programmes. Quantity assumptions in this area are based on published expert opinions. Quantification of the requirements at the country level is essential to strengthen these estimates.

**Table 4:** Percentage of countries with available data for selected variables, by income level

Country	Health care facilities, %	International salaries, %	Energy price (kWh USD 2010), %	Water price (per cubic metre USD 2010), %
High-income	60	60	62	74
Upper middle-income	68	50	27	52
Lower middle-income	75	25	8	41
Low-income	86	24	0	38

We were unable to use published costing data to further develop quantity assumptions and unit prices due to the general lack of detail provided in such studies. The majority of published costing studies present unit costs, without separating quantity assumptions and unit prices. In order to understand what drives differences in prices across countries we must understand the quantities and prices making up these unit costs. Reporting standards in this area towards greater disaggregation of cost data would drive improvements in programme costing analysis. Similarly, despite common consensus on the increasing marginal costs of implementing interventions in the final phases of scale up [35], there is a dearth of literature supporting this quantitatively [36].

## Conclusions

Future costing studies should consider the benefits of integrated approaches to programme delivery [37]. A stronger focus on the operational costs of the health system at varying levels of capacity and in situations where interventions are at differing coverage levels would be desirable to support improvements in future costing work, and would help support the transferability of findings from one disease area to others. More effort should be also be geared towards evaluating differences in the quality of service delivery corresponding to varying levels of programme investments in order to inform quantity assumptions.

## Footnotes

1 Defined as the application of organized knowledge and skills in the form of devices, medicines, vaccines, procedures and systems developed to solve a health problem and improve quality of lives.

2 WHO geographical regions are described here http://www.who.int/about/regions/en/.
